# Cardiovascular magnetic resonance as an initial screening tool in individuals with SLE and chest pain

**DOI:** 10.1136/lupus-2025-001652

**Published:** 2025-11-10

**Authors:** Isak Samuelsson, Simon Thalén, Giorgia Grosso, Magnus Lundin, Henrik Engblom, Peder Sörensson, Iva Gunnarsson, Martin Ugander, Elisabet Svenungsson

**Affiliations:** 1 Division of Rheumatology, Department of Medicine Solna, Karolinska Institutet, Stockholm, Sweden; 2 Department of Cardiology, Danderyd University Hospital, Stockholm, Sweden; 3 Clinical Physiology – Marcus Carlsson’s Research Group, Department of Molecular Medicine and Surgery, Karolinska University Hospital, Karolinska Institutet, Stockholm, Sweden; 4 Department of Radiology, Stanford University, Stanford, California, USA; 5 Division of Rheumatology, Department of Medicine Solna, Karolinska University Hospital, Karolinska Institutet, Stockholm, Sweden; 6 Department of Clinical Physiology, Södersjukhuset AB, Stockholm, Sweden; 7 Clinical Physiology, Department of Clinical Sciences Lund, Skåne University Hospital, Lund University, Lund, Sweden; 8 Endothelial Dysfunction in Atherosclerotic Cardiovascular Disease – Research Group John Pernow, Department of Medicine Solna, Karolinska Institutet, Stockholm, Sweden; 9 Department of Cardiology, Karolinska University Hospital, Stockholm, Sweden; 10 University of Sydney, Sydney, New South Wales, Australia

**Keywords:** Lupus Erythematosus, Systemic, Magnetic Resonance Imaging, Cardiovascular Diseases, Microcirculation, Atherosclerosis

## Abstract

**Objective:**

Individuals with SLE commonly report chest pain or discomfort. We performed cardiovascular magnetic resonance (CMR) to differentiate coronary artery disease (CAD), coronary microvascular dysfunction (CMD), pericarditis and myocarditis in individuals with SLE who presented with chest symptoms. We also assessed the clinical utility of CMR.

**Methods:**

Adults with SLE were included if reporting chest pain or dyspnoea suggestive of cardiac involvement to a rheumatologist between 2018 and 2023. Individuals underwent CMR, including quantitative myocardial perfusion mapping at rest and during adenosine stress if not contraindicated. CAD, CMD, pericarditis and myocarditis were identified by CMR. Confirmatory investigations were performed when indicated. We reviewed medical files to assess if CMR led to altered medical treatment or invasive interventions.

**Results:**

Nineteen individuals with SLE (84% female) with a median age of 39 (IQR 31–55) years underwent CMR, of whom 14 (74%) were examined using adenosine stress. Symptoms prompting inclusion were pleuritic chest pain in 10/19 (53%), chest pain triggered by exercise or relieved by nitrates or rest in 2/19 (11%), other types of chest pain in 5/19 (26%) and dyspnoea suggestive of cardiac involvement in 2/19 (11%). CAD, CMD and pericarditis were diagnosed in 3/14 (21%), 2/14 (14%) and 3/19 (16%) individuals, respectively. None had myocarditis. CMR revealed no cause of chest symptoms in 12/19 (63%). The CMR results led to altered medical management in 6/19 (32%) individuals.

**Conclusions:**

This cross-sectional study highlights cardiac ischaemia as a cause of chest symptoms in SLE. Notably, CAD and CMD were together more common than pericarditis and myocarditis. CMR may aid early detection and treatment of these conditions, as it altered medical management in one-third of cases. Larger studies are needed to confirm our findings and prospectively evaluate the long-term prognostic impact of early CMR in symptomatic individuals with SLE.

**WHAT IS ALREADY KNOWN ON THIS TOPIC**

- Individuals with SLE have increased morbidity and mortality due to heart disease compared with the general population.
- Chest pain or discomfort in individuals with SLE is multifactorial, with causes including coronary artery disease (CAD), coronary microvascular dysfunction (CMD), pericarditis and myocarditis.

**WHAT THIS STUDY ADDS**

- In the present study, individuals with SLE and chest pain or dyspnoea suggestive of cardiac involvement were investigated using cardiovascular magnetic resonance (CMR) and diagnosed with CAD, CMD, pericarditis or myocarditis in 21%, 14%, 16% and 0% of cases, respectively.
- CMR facilitated early identification and initiation of disease-specific treatment in 32% of individuals investigated with CMR.

**HOW THIS STUDY MIGHT AFFECT RESEARCH, PRACTICE OR POLICY**

- This study emphasises consideration of cardiac ischaemia due to CAD and CMD in individuals with SLE presenting with chest symptoms. We demonstrate that CMR can be used as a screening investigation for early detection of heart disease in individuals with SLE and chest symptoms.
- This study provides a foundation for larger studies to confirm the results and evaluate the long-term prognostic impact of early CMR in individuals with SLE and chest symptoms.

## Introduction

Chest pain or discomfort is frequently reported by individuals with SLE.^1^ These symptoms may originate from various lung and musculoskeletal conditions. Chest symptoms may also be due to heart disease, which has been associated with a twofold to threefold increased risk of mortality in SLE.^2^ Early identification of cardiac causes of chest symptoms is thus important to initiate cardioprotective treatment to ameliorate symptoms and improve long-term prognosis when possible.

In clinical practice, chest symptoms are often considered to be due to pleuropericarditis, a clinical criterion for SLE classification.^3 4^ Although rare, lupus myocarditis is another possible cause of chest symptoms in this population.^5^ Additionally, SLE increases risk of ischaemic heart disease (IHD) independent of traditional cardiovascular risk factors.^6^,^9^ This underscores the importance of considering pericarditis, myocarditis and IHD in individuals with SLE who present with chest symptoms.

The high risk of IHD in SLE is generally assumed to be caused by accelerated coronary artery disease (CAD). This view is supported by several observations, including a meta-analysis of coronary CT angiography (CCTA) investigations.^10^ This study reported that coronary calcium scores greater than 0, that is, the cut-off for asymptomatic CAD, were more common in individuals with SLE compared with controls.^10^ Another study supporting this view studied individuals with SLE and matched comparators, who underwent invasive coronary angiography on clinical indications.^11^ Although individuals with SLE were on average 21 years younger than their controls, both groups had similar extent of CAD.^11^

Coronary microvascular dysfunction (CMD), another subtype of IHD, has recently gained increased attention in the general population.^12^ However, CMD is less studied in SLE. Few have investigated individuals with SLE and chest symptoms using advanced imaging techniques like stress cardiovascular magnetic resonance (CMR) or positron emission tomography (PET).^13 14^ These modalities detect CMD by measuring myocardial perfusion at rest and during pharmacological hyperaemic stress, with excellent agreement between the two.^15^ Previous studies using CMR or PET suggest that individuals with SLE have a higher risk of CMD compared with those without SLE,^13 14^ but more studies are warranted.

In conclusion, chest pain or discomfort is common in SLE and may originate from undetected heart disease, which is associated with increased mortality in this population.^2^ Pericarditis, CAD, CMD and myocarditis are important heart diseases to consider in individuals with SLE presenting with chest symptoms.^3^,^510 11 13 14^ However, data on the relative prevalence of these cardiac conditions are limited, making diagnostic work-up harder. Therefore, we assessed the frequencies of pericarditis, CAD, CMD and myocarditis in individuals with SLE presenting with chest symptoms, using CMR as a screening modality. We also evaluated the clinical utility of CMR, as indicated by whether it led to altered medical management or not.

## Methods

The frequencies of pericarditis, CAD, CMD and myocarditis were estimated in individuals with SLE and chest symptoms. These heart diseases were diagnosed using CMR together with clinical assessments performed by rheumatologists and cardiologists according to clinical routine. We assessed whether CMR results altered medical management or not, using medical records from follow-up visits.

### Study participants

Individuals aged ≥18 years with SLE were included if reporting chest pain or dyspnoea suggestive of cardiac involvement to a rheumatologist at Karolinska University Hospital in Stockholm, Sweden, between April 2018 and May 2023. SLE was defined according to the 1982 revised American College of Rheumatology (ACR)^3^ and/or the Systemic Lupus International Collaborating Clinics (SLICC) classification criteria.^16^

Included individuals underwent CMR. We excluded individuals with prior allergic reactions to gadolinium-based contrast agents, severe claustrophobia, an estimated glomerular filtration rate (GFR) ≤30 mL/min/1.73 m^2^ or any other contraindication to CMR related to in-dwelling ferromagnetic material or implants where CMR was otherwise contraindicated.

Contraindications to pharmacological stress with adenosine, including atrioventricular (AV) blocks II–III before or during pharmacological stress, asthma or chronic obstructive pulmonary disease, did not constitute exclusion criteria. These individuals underwent CMR but without pharmacological stress. Thus, study participants were not selected for adenosine stress based on chest pain type or clinical presentation; rather, the presence of contraindications to adenosine stress.

### CMR protocol

CMR was performed at 3 or 1.5 T according to a predefined protocol outlined in online supplemental figure S1. The CMR protocol included full-coverage cine imaging to assess ventricular volumes and function. Late gadolinium enhancement (LGE) was used to assess the presence of myocardial scarring and enhancement of the pericardium. Quantitative stress first-pass myocardial perfusion images were screened for perfusion defects. Quantitative stress perfusion maps were assessed for regional and global myocardial perfusion defects. Native T1 and T2 maps and extracellular volume maps were assessed for diffuse myocardial fibrosis, myocardial oedema and infiltrative cardiomyopathies. CMR image interpretation was performed in clinical routine by at least one experienced physician with European Association of Cardiovascular Imaging level III certification not blinded to the investigated individual’s medical history.

Pericarditis was evaluated according to Adler *et al*,^17^ with pericardial thickening or inflammation, as evidenced by LGE and/or oedema, considered positive. Myocarditis was evaluated according to the 2018 Lake Louise criteria with at least one T1-based criterion of non-ischaemic myocardial injury as well as T2-based signs of myocardial oedema considered positive.^18^

**Figure 1:**
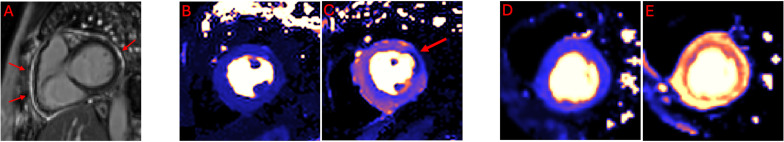
Three examples of pathologies in three different individuals with SLE. Late gadolinium enhancement imaging in short axis (A) reveals hyperenhancement of the pericardium (red arrows) indicative of pericarditis. Quantitative perfusion images in short axis at rest (B) and stress (C) reveal a regional perfusion defect (red arrow) indicative of obstructive coronary artery disease. Quantitative perfusion images in short axis at rest (D) and stress (E) with a modest perfusion increase during stress indicative of microvascular dysfunction.

CAD and CMD were evaluated according to Kotecha *et al*.^19^ A regional perfusion defect identified by quantitative perfusion mapping with a regional stress myocardial blood flow (MBF) ≤1.94 mL/g/min in a coronary territory, or, in the absence of a discrete regional defect, the presence of a visual perfusion defect, together with a global stress MBF ≤2.25 mL/g/min, was considered indicative of CAD. CMD was deemed present either when no regional or visual perfusion defect was observed but the global stress MBF was ≤2.25 mL/g/min, or when a regional perfusion defect was accompanied by a corresponding regional stress MBF >1.94 mL/g/min. CMR findings indicative of pericarditis, CAD and CMD are illustrated in figure 1.

### Definitions of the study outcomes

Individuals with CMR findings suggestive of CAD or CMD were referred to a cardiologist, while those with findings supporting pericarditis or myocarditis were followed up by a rheumatologist. Individuals with neither pericarditis, CAD, CMD nor myocarditis at CMR were classified as having chest symptoms of non-cardiac origin.

Presence of pericarditis, CAD, CMD and/or myocarditis was determined according to the treating cardiologist or rheumatologist at follow-up. The treating physician was informed of the CMR results and performed additional testing, such as invasive coronary or CT angiography, when clinically indicated rather than per protocol. We also assessed whether medical management, defined as invasive interventions or altered drug prescriptions, was changed during follow-up because of the CMR results. Retrospective medical file review was used for data collection on the study outcomes.

### Other variables

Retrospective medical file review was used to collect data on symptoms prompting inclusion categorised into angina-like chest pain, pleuritic chest pain, other types of chest pain and dyspnoea suggestive of cardiac involvement. Angina-like chest pain was defined as chest pain triggered by exercise and/or relieved by nitrates or rest. Pleuritic chest pain was defined as becoming worse with breathing or positional changes. Chest pain not meeting any of the previous two definitions was classified as other types of chest pain. Dyspnoea suggestive of cardiac involvement was defined as exercise-induced dyspnoea suggestive of cardiac ischaemia.

SLE disease duration, smoking status and comorbidities were collected using retrospective medical file review up until CMR. Reported comorbidities included previous cardiovascular disease, defined as previous CAD, stroke, transient ischaemic attack, aortic aneurysm or peripheral arterial disease. These comorbidities were also reported separately. Previous CAD was defined as a medical history of myocardial infarction (MI), unstable angina or chronic coronary syndrome. MI was defined as medical files consistent with the fourth universal definition of MI^20^ or CMR findings of previous MI at inclusion in those without any documented MI in their medical records. Further definitions of comorbidities are provided in online supplemental table S1.

Furthermore, we reported prevalence of previous venous thromboembolism and diabetes defined in online supplemental table S1, lupus nephritis according to kidney biopsies or Tan *et al*,^3^ secondary antiphospholipid syndrome (APS) as defined by Miyakis *et al*^21^ and secondary Sjögren’s syndrome as defined by Vitali *et al*.^22^ The time between inclusion and CMR was calculated using data from medical records.

Blood and urine samples were collected adjacent to CMR and analysed as per clinical routine at the Department of Clinical Chemistry, Karolinska University Hospital. Creatinine-based estimated GFRs were calculated according to the revised Lund-Malmö GFR estimating equation.^23^ ANA screening was performed using indirect immunofluorescence in Hep-2 cells (Immunoconcepts, Sacramento, California, USA). Antibodies towards double‐stranded DNA, nucleosomes, Smith (Sm), ribonucleoprotein (RNP) 68, Sm/RNP, ribosomal P, cardiolipin immunoglobulin G (IgG)/IgM, β_2_ glycoprotein 1 IgG/IgM, Sjögren’s syndrome antigen A (SSA)/Ro52, SSA/Ro60 and Sjögren’s syndrome antigen B were analysed in serum by BioPlex 2200 (Bio-Rad Laboratories, Hercules, California, USA).

Laboratory data and retrospective medical file review were used to assess disease activity according to the Systemic Lupus Erythematosus Disease Activity Index 2000 (SLEDAI-2K)^24^ and damage accrual according to the Systemic Lupus International Collaborating Clinics/American College of Rheumatology Damage Index (SLICC/ACR DI).^25^ Data on laboratory results, comorbidities and blood pressure were used to estimate traditional cardiovascular risk according to the European Society of Cardiology (ESC) and Visseren *et al*.^26^ Blood pressure and body mass index were measured at the time of CMR.

### Statistical analysis

The distributions of study variables were described using descriptive statistics, that is, median and IQR, or frequencies, as appropriate. No statistical testing was performed, as this study had limited power and was descriptive.

### Patient and public involvement

Neither individuals with SLE nor the public were involved in the design, conduct, reporting or dissemination plans of this study.

## Results

Nineteen individuals with SLE were included, of whom 16 (84%) were females. Median age and SLE disease duration at CMR were 39 (IQR 31–55) and 11 (IQR 4–13) years, respectively. Included individuals had a median SLICC/ACR DI of 1 (IQR 0–1) and a median SLEDAI-2K score of 5 (IQR 4–6). Seven out of 19 (37%) had a history of lupus nephritis, while secondary APS and secondary Sjögren’s syndrome were less common, corresponding to 1 of 19 (5%) and 1 of 19 (5%) individuals, respectively (table 1).

**Table 1:** Characteristics of included individuals with SLE at CMR

Variable	Measure*	Number of observations with complete data
Female, n (%)	16 (84)	19/19
Age, years	39 (31–55)	19/19
Disease duration, years	11 (4–13)	19/19
SLICC/ACR DI†	1 (0–1)	19/19
Previous lupus nephritis‡, n (%)	7 (37)	19/19
Sjögren’s syndrome§, n (%)	1 (5)	19/19
APS¶, n (%)	1 (5)	19/19
Previous VTE**, n (%)	4 (21)	19/19
Prednisolone equivalent dose, mg	5 (5–15)	19/19
HCQ treatment, n (%)	15 (79)	19/19
Symptoms prompting inclusion††, n (%)		19/19
Pleuritic chest pain	10 (53)	
Angina-like chest pain	2 (11)	
Other types of chest pain	5 (26)	
Dyspnoea suggestive of cardiac involvement	2 (11)	
SLEDAI-2K‡‡	5 (4–6)	19/19
C-reactive protein, mg/L	3 (1–4)	17/19
Sedimentation rate, mm	27 (15–50)	17/19
Plasma albumin, g/L	34 (30–37)	17/19
Platelet count, ×10^9^/L	320 (220–360)	17/19
Leucocyte count, ×10^9^/L	6 (4–7)	17/19
Lymphocyte count, ×10^9^/L	1 (1–1.5)	17/19
Plasma creatinine, µmol/L	75 (61–88)	19/19
Albumin-creatinine ratio in urine, mg/mmol	3 (1–19)	15/19
ANA, n (%)	12 (100)	12/19
Anti-dsDNA, n (%)	10 (59)	18/19
Anti-nucleosome, n (%)	14 (82)	17/19
Anti-Sm, n (%)	4 (24)	17/19
Anti-SmRNP, n (%)	7 (41)	17/19
Anti-RNP68, n (%)	3 (18)	17/19
Anti-ribosomal P, n (%)	2 (12)	17/19
Anti-CL IgG, n (%)	2 (12)	17/19
Anti-CL IgM, n (%)	0 (0)	16/19
Anti-β_2_GP1 IgG, n (%)	2 (12)	17/19
Anti-β_2_GP1 IgM, n (%)	0 (0)	16/19
Lupus anticoagulant, n (%)	2 (14)	14/19
Anti-SSA/Ro52, n (%)	9 (53)	17/19
Anti-SSA/Ro60, n (%)	12 (71)	17/19
Anti-SSB, n (%)	6 (35)	17/19
C1q, mg/L	100 (72–121)	14/19
C3, g/L	0.8 (0.6–1)	16/19
C4, g/L	0.1 (0.1–0.2)	13/19
C3d, mg/L	7 (6–8)	15/19

* Data are reported as absolute counts (frequencies) or medians (IQRs) as appropriate.

† Calculated according to Gladman *et al*.^25^

‡ Defined according to biopsy material or Tan *et al*.^3^

§ Defined according to Vitali *et al*.^22^

¶ Defined according to Miyakis e*t al*.^21^

** Defined as pulmonary embolism or deep vein thrombosis.

†† Chest pain was categorised into pleuritic (worse with breathing or positional), angina-like (triggered by exercise and/or relieved by nitrates/rest), other types of chest pain (neither angina-like nor pleuritic chest pain) and dyspnoea suggestive of cardiac ischaemia.

‡‡ Calculated according to Gladman *et al*.^24^

APS, antiphospholipid syndrome; CL, cardiolipin; CMR, cardiovascular magnetic resonance; dsDNA, double-stranded DNA; HCQ, hydroxychloroquine; IgG, immunoglobulin G; IgM, immunoglobulin M; RNP, ribonucleoprotein; SLEDAI-2K, Systemic Lupus Erythematosus Disease Activity Index 2000; SLICC/ACR DI, Systemic Lupus International Collaborating Clinics/American College of Rheumatology Damage Index; Sm, Smith; SSA, Sjögren’s syndrome antigen A; SSB, Sjögren’s syndrome antigen B; VTE, venous thromboembolism; β_2_GP1, β_2_ glycoprotein 1.

Pleuritic chest pain was the most common symptom at inclusion, reported by 10 of 19 (53%) individuals. Other symptoms prompting inclusion were less common. Angina-like chest pain was present in 2 of 19 (11%) individuals, while other types of chest pain were reported by 5 of 19 (26%) individuals. Dyspnoea suggestive of cardiac involvement was present in 2 of 19 individuals (11%). Further baseline characteristics are presented in table 1.

Assessment of traditional cardiovascular risk according to ESC and Visseren *et al*^26^ followed a bimodal distribution. Out of 17 of the 19 (89%) individuals with complete data, 10 (59%) were classified as low to moderate risk. One (6%) individual was classified as high risk. Six (35%) individuals were classified as very high risk, of whom three (16%) had CAD prior to inclusion. Traditional cardiovascular risk factors at inclusion are presented in more detail in table 2.

**Table 2:** Traditional cardiovascular risk factors in individuals with SLE at CMR

Variables	Measure*	Number of observations with complete data
Risk of CVD according to ref, ^26^ n (%)†		17/19
Low to moderate risk	10 (59)	
High risk	1 (6)	
Very high risk	6 (35)	
CVD‡, n (%)	5 (26)	19/19
CAD§, n (%)	3 (16)	19/19
Creatine-based eGFR¶, mL/min/1.73 m^2^	77 (69–93)	19/19
Type 1 or type 2 diabetes, n (%)	0 (0)	19/19
Smoking status, n (%)		18/19
Current	0 (0)	
Previous	9 (50)	
Never	9 (50)	
Systolic BP, mm Hg	120 (110–130)	19/19
Diastolic BP, mm Hg	79 (71–83)	19/19
Antihypertensive treatment, n (%)	9 (47)	19/19
BMI, kg/m^2^	25 (20–28)	19/19
Total cholesterol, mmol/L	4.7 (4.2–5.0)	14/19
LDL, mmol/L	2.6 (2.2–2.7)	13/19
HDL, mmol/L	1.5 (1.2–1.7)	14/19
Triglycerides, mmol/L	1.3 (1.0–1.9)	14/19
Cholesterol-lowering treatment, n (%)	4 (21)	19/19

* Data collected through retrospective medical file review and blood samples drawn adjacent to the CMR are reported as absolute counts (frequencies) or medians (IQRs) as appropriate.

† Traditional cardiovascular risk was estimated according to Visseren *et al*.^26^

‡ Defined as myocardial infarction, unstable angina, chronic coronary syndrome, stroke, transient ischaemic attack, aortic aneurysm or peripheral arterial disease.

§ Defined as myocardial infarction, unstable angina or chronic coronary syndrome.

¶ According to the revised Lund-Malmö GFR estimating equation.^23^

BMI, body mass index; BP, blood pressure; CAD, coronary artery disease; CMR, cardiovascular magnetic resonance; CVD, cardiovascular disease; eGFR, estimated glomerular filtration rate; GFR, glomerular filtration rate; HDL, high-density lipoprotein; LDL, low-density lipoprotein.

The median time between inclusion and CMR was 10 (IQR 4–23) days. Pharmacological stress was used in 14/19 (74%) individuals. Reasons for not performing or interrupting pharmacological stress in enrolled individuals included asthma (n=2), AV block during infusion (n=1), miscommunication in the referral for CMR (n=1) and failure to obtain vascular access (n=1), which also hindered the administration of the gadolinium-based contrast agent.

CAD and CMD were together more common than pericarditis and myocarditis, corresponding to 3 of 14 (21%), 2 of 14 (14%), 3 of 19 (16%) and 0 of 19 (0%) individuals, respectively. Collectively, pericarditis, CAD and/or CMD were found in 7 of 19 (37%) individuals. Note that these diseases were not mutually exclusive, as one individual had both CAD and pericarditis. More CMR-related estimates not reported as the primary outcome are presented in table 3.

**Table 3:** Detailed data from CMR investigations

CMR variable	Measure*	Number of observations with complete data
LVEDV, mL	148 (125–157)	19/19
LVEDVI, mL/m^2^	78 (68–87)	19/19
LVESV, mL	57 (50–71)	19/19
LVESVI, mL/m^2^	32 (27–53)	19/19
SV, mL	79 (71–93)	19/19
SVI, mL/m^2^	44 (36–51)	19/19
LVM, g	103 (93–121)	19/19
LVMI, g/m^2^	60 (53–67)	19/19
LVEF, %	59 (53–64)	19/19
ECV, %	28 (25–29)	19/19
Myocardial T1, ms†	1316 (1286–1357)	17/19
Myocardial T2, ms†	43 (43–46)	11/19
Rest perfusion, mL/min/g	0.9 (0.9–1.1)	14/19
Stress perfusion, mL/min/g	2.7 (2.5–3.0)	14/19
Myocardial LGE, %	4 (22)	18/19
Pericardial LGE, %	1 (6)	18/19
Pericardial effusion >5 mm, %	3 (17)	19/19

* Data are reported as absolute counts (frequencies) or medians (IQRs) as appropriate.

† T1/T2 values were acquired at a field strength of 3 T.

CMR, cardiovascular magnetic resonance; ECV, extracellular volume; LGE, late gadolinium enhancement; LVEDV, left ventricular end-diastolic volume; LVEDVI, left ventricular end-diastolic volume index; LVEF, left ventricular ejection fraction; LVESV, left ventricular end-systolic volume; LVESVI, left ventricular end-systolic volume index; LVM, left ventricular mass; LVMI, left ventricular mass index; SV, stroke volume; SVI, stroke volume index.

Two of the three individuals with prior CAD were diagnosed with recurrent CAD after being investigated with stress CMR. One had a previous MI successfully treated with percutaneous coronary intervention (PCI) of the left anterior coronary artery. Despite previous PCI and medical treatment, coronary angiography prompted by stress CMR revealed significant coronary obstruction affecting all three major coronary arteries. This finding resulted in coronary bypass grafting and adjusted medical therapy (individual 4, table 4).

**Table 4:** Individual-level data for individuals with pericarditis, CAD or CMD

Index	Age group (years)	Sex	SLE duration (years)	Previous CAD	CMR findings	Global perfusion defect	Regional perfusion defect	Coronary angiography findings*	Clinical conclusion†	Medical intervention
1	20–25	Female	12	No	CMD	Yes	No	Not performed	CMD	Medications altered
2	40–45	Male	6.5	CMR-verified MI at inclusion, but no CAD at coronary angiography 4 months prior to inclusion	Previous MI and CMD or balanced CAD in all three major coronary arteries	Yes	Yes	Complete blockage in a side branch of the LCx and CMD in the main LCx‡	CAD	Medications altered
3	55–60	Female	0.1	No CAD	Pericardial effusion	Not tested	Not tested	Not performed	Pericarditis	Medications altered
4	55–60	Female	12	Previous MI with PCI of the LAD	Reduced LVEF, general hypokinesia and a subendocardial perfusion rest defect. Questionable stress effect.	Inconclusive	Yes	Significant atherosclerosis affecting all major coronary arteries	CAD	CABG and medications altered
5	55–60	Female	27	No	Pericarditis with active inflammation. Stress-induced ischaemia in the RCA territory.	No	Yes	Not performed	CAD and pericarditis	Medications altered
6	55–60	Female	2.3	No	Pericarditis with active inflammation	No	No	Not performed	Pericarditis	No change
7	55–60	Female	13	No	CMD	No	Yes	Not performed	CMD	Medications altered

* No individual underwent CT angiography post-CMR.

† According to the treating physician.

‡ As indicated by an index of microcirculatory resistance of 25.

CABG, coronary artery bypass grafting; CAD, coronary artery disease; CCS, chronic coronary syndrome; CMD, coronary microvascular dysfunction; CMR, cardiovascular magnetic resonance; LAD, left anterior descending artery; LCx, left circumflex artery; LVEF, left ventricular ejection fraction; MI, myocardial infarction; PCI, percutaneous coronary intervention; RCA, right coronary artery.

A second individual was classified as having prior CAD based on CMR findings which indicated a previous MI, despite no documented MI in the medical records and no significant atherosclerosis on a coronary angiography performed 4 months earlier. Myocardial perfusion mapping showed decreased perfusion, and repeat coronary angiography revealed an index of microcirculatory resistance of 25 in the main left circumflex artery (LCx) and a chronically occluded marginal branching from the LCx. This individual was diagnosed with CAD and prescribed several new medications due to this finding (individual 2, table 4).

We also sought to evaluate CMR as a screening modality, which led to changed medical management in six of seven individuals with CAD, CMD and/or pericarditis. All individuals with CAD or CMD underwent changes in medical therapy due to altered drug prescriptions, but also coronary bypass grafting in one case. Two out of three individuals with pericarditis underwent changes in prescribed medical treatment (table 4).

Twelve individuals (83% female) with a median age of 37 (IQR 30–46) years and a median SLE duration of 11 (IQR 7–14) years showed no signs of CAD, CMD or perimyocarditis on CMR. Consequently, they did not undergo any further diagnostic work-up or therapeutic interventions. Although T2 mapping was not performed in all individuals, none of those with missing T2 data had elevated T1 values nor missing data regarding T1 measurements. Myocarditis was therefore excluded in accordance with the 2018 Lake Louise criteria. Four of the 12 individuals did not undergo adenosine stress testing due to asthma (n=1), AV block during adenosine infusion (n=1), dysfunctional peripheral lines (n=1) or referral error (n=1). As a result, CAD and CMD could not be completely ruled out in these individuals.

## Discussion

This study assessed the frequencies of pericarditis, CAD, CMD and myocarditis in 19 individuals with SLE who presented with chest pain or dyspnoea suggestive of cardiac involvement. We performed CMR and used sequential clinical follow-up data obtained from visits with cardiologists or rheumatologists. Notably, cardiac ischaemia due to CAD or CMD was more common than pericarditis and myocarditis in our study sample. The CMR results, together with clinically indicated follow-up, led to altered medical management in 6 of 19 individuals, supporting CMR as a screening modality in individuals with SLE presenting with chest symptoms.

In clinical practice, chest symptoms in individuals with SLE are often attributed to pleuropericarditis or musculoskeletal conditions. However, the present study highlights cardiac ischaemia due to CAD or CMD, rather than pericarditis, as an important cardiac cause of chest symptoms in SLE. Our results align with previous studies in which SLE has been identified as a risk enhancer for CAD^10 11^ and CMD,^13 14^ further emphasising the need to consider cardiac ischaemia in individuals with SLE. In this limited sample, we observed no cases of myocarditis. This is not surprising since myocarditis is fairly rare among adults with SLE.^5^

This study is the first to use fully quantitative stress CMR to assess CAD and CMD in SLE. However, a few studies are available for comparison.^13 14^ Ishimori *et al*^14^ used semiquantitative stress CMR and CCTA to investigate 20 women with SLE and chest pain, as well as 10 asymptomatic female comparators. The average age (41±11 years) and gender distribution in the SLE group were like the present study. However, in contrast to Ishimori *et al*,^14^ who excluded all individuals with established CAD or arrhythmias, we included individuals with SLE and chest symptoms regardless of previous diagnoses. Furthermore, their study participants were living in the USA, while ours were Swedish residents, enrolled approximately a decade later. Thus, differences in genetics and environmental exposures such as medical treatments, which have evolved substantially during the last decade,^27^ may account for substantial variation between study samples.

Ishimori *et al*^14^ reported that SLE was associated with CMD, which was diagnosed in 8 of 18 (44%) females with SLE. None of these had >50% stenosis on CCTA.^14^ In our study, 2 of 14 (14%) individuals had CMD, out of which none had previous CAD. This represents a lower frequency compared with Ishimori *et al*.^14^ However, given the differences in target populations, study designs and the limited sample sizes in both studies, a quantitative comparison is not applicable. Nevertheless, both studies support CMD as a considerable cause of chest symptoms in SLE.

Weber *et al*^13^ also studied CMD in SLE. These authors included individuals with SLE and matched comparators, all of whom had chest pain or dyspnoea prompting semiquantitative PET myocardial perfusion imaging. Matching was based on age, sex and traditional cardiovascular risk factors. After the exclusion of individuals with obstructive CAD and left ventricular systolic dysfunction, 42 individuals with SLE and 69 comparators were included. Subjects with SLE were similar regarding gender distribution (97% female) but were on average older (61±0.5 years) relative to the present study. CMD was diagnosed in 24 of 42 (57%) individuals with SLE and was significantly more common compared with controls.^13^ Collectively, the present study, together with previous studies,^13 14^ supports CMD as a considerable cause of chest symptoms in SLE.

It is not surprising that we identified individuals with symptomatic CAD. Accelerated atherosclerosis, including CAD, has been studied in SLE for more than 20 years, but most commonly in asymptomatic subjects.^10 11 28^ Although our study is too small to draw any conclusions regarding cardiovascular risk factors, SLE is an established risk factor for IHD independent of traditional cardiovascular risk.^6^,^9^ Disease-specific factors matter, including long^8 29^ and short^8 9^ disease duration and older age at diagnosis.^7 8^

Subsets of individuals with SLE carry specific risk factors that confer additional cardiovascular risk, such as antiphospholipid antibodies^8 30^ and nephritis with reduced GFR.^31 32^ Therapies for SLE also play a role. Long-term corticosteroids raise cardiovascular risk,^33^ whereas hydroxychloroquine appears protective in some studies.^34^ Because SLE is heterogeneous, cardiovascular findings from any single small cohort—including ours—should not be extrapolated to all individuals; each individual’s pericarditis, CAD, CMD and myocarditis risk profile is unique.

Diagnosing chest pain or discomfort in SLE may be clinically challenging because there is yet no established way to determine pretest probability of pericarditis, CAD, CMD and myocarditis in this population. We demonstrate that using stress CMR as a screening modality in individuals with SLE presenting with chest symptoms can aid clinicians in their assessments and guide further investigations. While transthoracic echocardiography excels in availability, provides a rapid haemodynamic assessment and excludes significant pericardial effusion, CMR is more informative. It is increasingly available and offers a single, non-invasive, radiation-free examination that can differentiate between major cardiac complications of SLE. CMR enables comprehensive evaluation of IHD,^35^ pericarditis^36^ and myocarditis^18^ and has shown promise in differentiating CAD and CMD.^19^ However, in ambiguous cases, invasive coronary angiography providing measurements of epicardial stenoses/occlusions and microcirculatory pathology may be necessary to separate CAD from CMD.^12^

Moreover, in the diverse and heterogeneous SLE population, the high negative predictive value of CMR significantly helps rule out these complications,^37^ thus preventing individuals from unnecessary downstream testing and invasive procedures. In conclusion, due to its advantages, it is not surprising that CMR emerged as an important screening tool, altering medical management in approximately one-third of the individuals in our study. The long-term prognostic impact of early CMR in SLE remains to be determined in future studies.

### Strengths and limitations

This study is the first real-life clinical evaluation of individuals with SLE and chest symptoms using a comprehensive, state-of-the-art CMR protocol with fully quantitative stress myocardial perfusion. The CMR protocol provides non-invasive, radiation-free, robust and operator-independent assessments of pericarditis, CAD, CMD and myocarditis. In addition, all individuals with SLE met the 1982 ACR^3^ or the SLICC classification criteria for SLE,^16^ making the SLE diagnosis robust.

The present study is limited by the small sample size, which impacts the precision and generalisability of the observed outcome data. Assessment of SLE duration as a risk stratifier was not performed, as subdivision by disease duration and outcome would have yielded strata too small for robust statistical analysis. Individuals with SLE were not randomly assigned to CMR or standard of care, which would have been ideal for assessing the clinical value of CMR in this group.

CAD and CMD could not be assessed in 5 of 19 individuals due to asthma (n=2), AV block during adenosine infusion (n=1), malfunctioning peripheral lines (n=1) and referral error (n=1). Instead of excluding these individuals, they underwent CMR without adenosine stress to preserve precision and generalisability of pericarditis and myocarditis estimates, as our study was already impacted by a small sample size. This approach provided a realistic reflection of the real-world clinical utility of CMR in SLE, where similar practical limitations frequently occur.

The study has also limitations related to potential misclassification of the study outcomes. While the individuals with SLE were recruited and included prospectively, most data were collected retrospectively, which inherits risks regarding accuracy. Furthermore, performing invasive coronary angiography or CCTA at follow-up per protocol, rather than only when clinically indicated, would have been preferable to differentiate CMD and CAD. Additionally, CMR was performed at a median of 10 days after referral. During this time, some individuals had already begun corticosteroid treatment for suspected pericarditis, potentially leading to an underestimation of the frequency of pericarditis detected in the present study.

## Conclusions

This study highlights the importance of considering cardiac ischaemia, namely CAD and CMD, in individuals with SLE and chest symptoms. In this study, CMR facilitated early identification and guided follow-up investigations and treatment of these cardiac conditions, known to be major causes of morbidity and mortality in SLE. Larger studies are needed to confirm our findings and to prospectively evaluate the long-term prognostic impact of early CMR in symptomatic individuals with SLE.

## Supplementary material

10.1136/lupus-2025-001652 online supplemental file 1

## Data Availability

Data are available upon reasonable request.
