# Supplementary material for: Cardiovascular magnetic resonance as an initial screening tool in individuals with SLE and chest pain
Source: Lupus Sci Med. 2025 Nov 10;12(2):e001652. doi: 10.1136/lupus-2025-001652 (PMC12603706; doi:10.1136/lupus-2025-001652)
Supplement: online supplemental file 1 [file lupus-12-2-s001.docx]

**SUPPLEMENTARY MATERIAL**

**Table of Contents**1. Supplementary Table S1 describing how comorbidities and traditional cardiovascular risk prior to cardiovascular magnetic resonance (CMR) were defined and how data were collected.

2. A detailed description of the CMR protocol used in this study.

3. Supplementary Figure S1 providing a visual overview of the CMR protocol.

4. References

**Table S1 – Definitions and data collection of comorbidities and traditional cardiovascular risk prior to cardiovascular magnetic resonance**

| **Variable name** | **Definitions and data collection** |
| --- | --- |
| Cardiovascular disease (CVD) | MI, UA, CCS, stroke, TIA, AA or PAD. See definitions below. |
| Coronary artery disease | MI, UA or CCS. See definitions below. |
| Myocardial infarction (MI) | Type 1-2 MIs according to Thygesen, et al. (1) were retrospectively identified using medical records. In addition, silent MIs, defined as the absence of documented previous MIs in medical records but with CMR evidence of a prior MI at inclusion, was classified as MI. |
| Unstable angina (UA) | UA according to Byrne, et al. (2) prompting ICA, PCI or CABG was required for retrospective identification of UA, using medical records |
| Chronic coronary syndrome (CCS) | CCS according to Vrints, et al. (3) prompting ICA, PCI or CABG or disease specific treatment (anti-ischemic or antiplatelet drugs as described in (3)) was required for retrospective identification of UA, using medical records. |
| Stroke | Both ischemic and hemorrhagic stroke were considered, but supporting evidence from diagnostic modalities (CT, MRI) or disease specific treatment (antiplatelet agents, thrombolytic therapy or thrombectomy) were required for retrospective identification of stroke, using medical records. |
| Transient ischemic attack (TIA) | In addition to classic symptoms of TIA, supporting evidence from MRI or prescription of antiplatelet drugs were required for retrospective identification of TIA, using medical records. |
| Aortic aneurysm (AA) | AA was identified using retrospective medical file review given existing documentation from diagnostic modalities (US, DT, MRI) or disease specific intervention (endovascular or surgical interventions). |
| Peripheral arterial disease (PAD) | All types of PADs were considered, but supporting evidence from diagnostic modalities (CT, DUS, invasive angiography or toe pressure) or disease specific treatment (antiplatelet agents, PTA or surgery including bypass grafting) were required for retrospective identification of PAD, using medical records. |
| Venous thromboembolism (VTE) | Pulmonary embolism and/or deep vein thrombosis were classified as VTE but evidence from diagnostic modalities (US, CT or DUS depending on VTE location) or disease specific treatment (LMWH, unfractioned heparin, OAC or other anti-thrombolytic treatments or interventions) were required for retrospective identification of VTE, using medical file review. |
| Antiphospholipid Syndrome | According to Miyakis, et al. (4). Identified retrospectively using medical records. |
| Sjögren’s syndrome | According to Vitali, et al. (5). Identified retrospectively using medical records. |
| Type 1 or 2 diabetes | Either laboratory evidence according to American Diabetes Association (6) or disease specific treatments (insulin or other antidiabetic drugs) were required for retrospective identification of diabetes, using medical files. |
| Lupus nephritis (LN) | LN was identified through retrospective medical file review given supporting evidence from renal biopsies or cases with proteinuria and/or cellular cast as described by Tan, et al. (7). |
| Traditional cardiovascular risk | Traditional cardiovascular risk was estimated in several steps:   1. Several comorbidities were identified using retrospective medical file review, including chronic kidney disease (eGFR <60 mL/min/1.7 or ACR >300 mg/mmol), familial hypercholesterolemia (according to the Dutch Clinical Lipid Network criteria and further described in (8)), type 1 or 2 diabetes mellitus (see definition above) and/or established CVD (see (8) and CVD definition above). Cardiovascular risk was then estimated according to Table 4 in (8). 2. In individuals without any of the above-mentioned comorbidities, cardiovascular risk was estimated using the SCORE2 or SCORE2-OP for populations at moderate CVD risk as described in (8). |

Abbreviations: ACR; albumin-to-creatinine-ratio; ICA, invasive coronary angiography; CABG, coronary artery bypass grafting; CMR, cardiovascular magnetic resonance; CT, computed tomography; DUS, duplex ultrasound; eGFR, estimated glomerular filtration rate; LMWH, low molecular weight heparin; MRI, magnetic resonance imaging; PCI, percutaneous coronary intervention; PTA, percutaneous transluminal angioplasty; OAC, oral anticoagulants; SCORE2, systematic coronary risk estimation 2; SCORE2-OP, systematic coronary risk estimation 2-older persons; US, ultrasound.

**Cardiovascular magnetic resonance imaging parameters**

CMR was performed at 3 Tesla (MAGNETOM Skyra, Siemens Healthcare, Erlangen, Germany) or 1.5 Tesla (MAGNETOM Aera, Siemens Healthcare, Erlangen, Germany). A standard phased-array, 18-channel body coil was used. All investigated individuals were examined in the supine position. An overview of the CMR protocol is provided in supplementary Figure S1.

Contrast-enhanced cine imaging was performed using retrospective ECG-gated balanced steady-state free precession (SSFP) and images acquired in full coverage short-axis slices and three long-axis slices to enable assessment of left ventricular volumes, ejection fraction, myocardial mass and the presence of pericardial effusion with the SyngoVia VA30 software (Siemens, Erlangen, Germany). Body surface area was calculated using the Mosteller formula (9), and volumetric measurements and myocardial mass indexed accordingly. Typical imaging parameters were flip angle 68°, pixel size 1.4 × 1.9 mm^2^, slice thickness 8.0 mm, echo time (TE)/repetition time (TR) 1.19 ms/37.05 ms, image matrix size 256 × 144, and field of view (FOV) of 360 × 270 mm^2^.

Myocardial perfusion imaging was performed using a first-pass perfusion sequence in three short-axis slices during systole (basal, mid-ventricular, apical). The sequence used an accelerated low-resolution fast low-angle shot (FLASH) to calculate the arterial input function and achieve linearity between the ventricular blood signal and the contrast agent concentration. A high-resolution SSFP readout was used to estimate myocardial perfusion. Typical imaging parameters were: flip angle 50°, slice thickness 8.0 mm, TE/TR 1.04 ms/2.5 ms, bandwidth 1085 Hz/pixel, FOV 360 × 270 mm^2^ and saturation delay/trigger delay 105/40 ms. Images were acquired during 90 heartbeats at rest and three minutes after initiation of pharmacological stress infusion with adenosine (Adenosin, Life Medical AB, Stockholm, Sweden, 140 µg/kg/min) following an intravenous bolus of a gadolinium-based contrast agent (0.05 mmol/kg, gadobutrol, Gadovist, Bayer AB, Berlin, Germany). Quantitative perfusion (ml/min/g) maps of the myocardium were generated inline using Gadgetron perfusion mapping software, and assuming a bi-distributed tissue exchange model (10).

T1-mapping was performed in three short-axis slices and three long-axis slices using a modified look-locker inversion (MOLLI, 5s(3s)3s) recovery prototype sequence. Typical imaging parameters were single shot SSFP in end-diastole, flip angle 35°, pixel size 1.4 × 1.9 mm^2^, slice thickness 8.0 mm, imaging duration 167 ms, TE/TR 1.12 ms/2.7 ms, image matrix 256 × 144 and FOV 360 × 270 mm^2^. Extracellular volume maps at rest were generated from native T1-maps and post-contrast T1-maps and calibrated by the hematocrit from venous blood sampling (11).

T2-mapping was performed in three short-axis slices and three long-axis slices using a T2-prepared sequence. Typical imaging parameters were TE/TR 1.06 ms/2.49 ms, flip angle 70°, pixel size 1.4 × 1.9 mm^2^, slice thickness 8.0 mm, acquisition window 700 ms, TD 483 ms and image matrix 256 × 144.

Late gadolinium enhancement (LGE) imaging was performed in three short-axis slices and three long-axis slices using a motion-corrected phase-sensitive inversion recovery sequence. Images were acquired at least 10 min after a total contrast injection volume of 0.2 mmol/kg in the same short- and long-axis imaging planes as cine imaging and assessed for late gadolinium enhancement in the myocardium and pericardium. An inversion time scout was used to determine the specific inversion time of the investigated individual's myocardium, nulling the myocardial signal during subsequent imaging. Typical imaging parameters were TE/TR 3.4 ms/7.8 ms, FOV 360 mm × 270 mm, image matrix 256 × 192 and a slice thickness of 8 mm for a spatial resolution of 1.4 x 1.4 x 8 mm^3^.


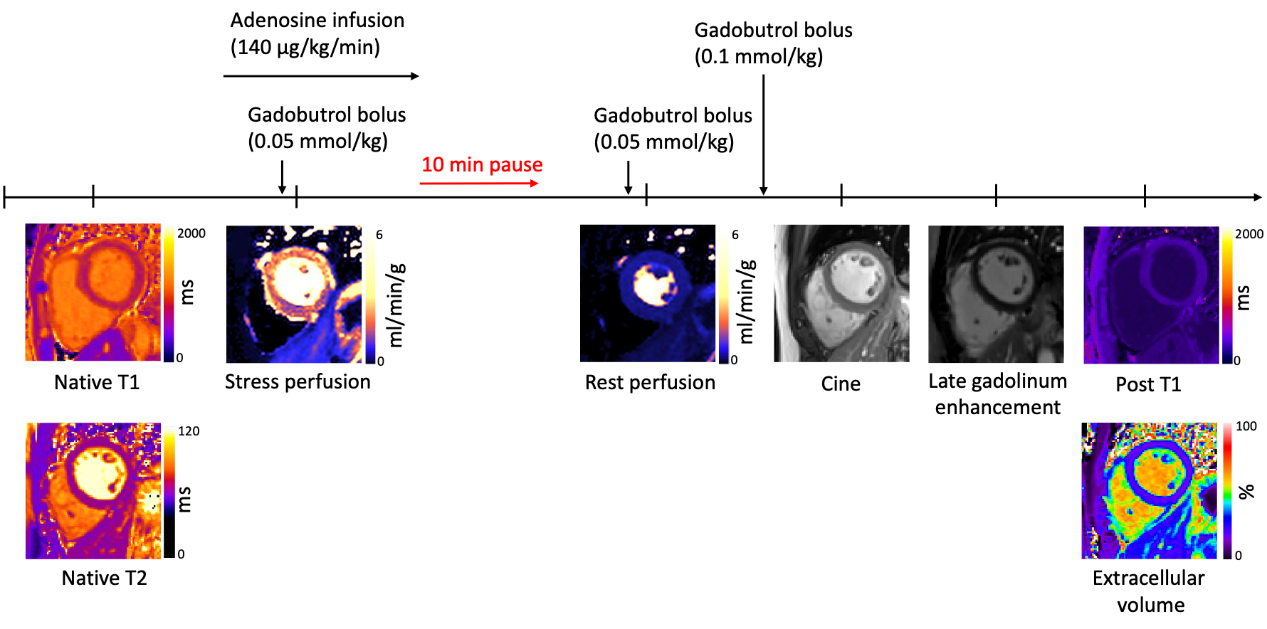


**Supplementary Figure S1 – An overview of the cardiovascular magnetic resonance protocol.** Native T1 and T2 maps were acquired first. After three minutes of adenosine infusion, stress first-pass perfusion maps were obtained using a 0.05 mmol/kg Gadobutrol bolus. Adenosine was then stopped, and after a 10-minute pause to allow for contrast equilibrium, rest perfusion maps were acquired using a second 0.05 mmol/kg Gadobutrol bolus. An additional 0.1 mmol/kg Gadobutrol bolus was then given, reaching a total dose of 0.2 mmol/kg. Standard cine images were acquired next, followed by late gadolinium enhancement images and post-contrast T1 maps.

**References**1. Thygesen K, Alpert JS, Jaffe AS, Chaitman BR, Bax JJ, Morrow DA, et al. Fourth Universal Definition of Myocardial Infarction (2018). Circulation. 2018;138(20):e618-e51.
2. Byrne RA, Rossello X, Coughlan JJ, Barbato E, Berry C, Chieffo A, et al. 2023 ESC Guidelines for the management of acute coronary syndromes: Developed by the task force on the management of acute coronary syndromes of the European Society of Cardiology (ESC). European heart journal. 2023.
3. Vrints C, Andreotti F, Koskinas KC, Rossello X, Adamo M, Ainslie J, et al. 2024 ESC Guidelines for the management of chronic coronary syndromes. European heart journal. 2024;45(36):3415-537.
4. Miyakis S, Lockshin MD, Atsumi T, Branch DW, Brey RL, Cervera R, et al. International consensus statement on an update of the classification criteria for definite antiphospholipid syndrome (APS). Journal of thrombosis and haemostasis : JTH. 2006;4(2):295-306.
5. Vitali C, Bombardieri S, Jonsson R, Moutsopoulos HM, Alexander EL, Carsons SE, et al. Classification criteria for Sjögren's syndrome: a revised version of the European criteria proposed by the American-European Consensus Group. Annals of the rheumatic diseases. 2002;61(6):554-8
6. American Diabetes Association. 2. Classification and Diagnosis of Diabetes: Standards of Medical Care in Diabetes-2018. Diabetes care. 2018;41(Suppl 1):S13-s27.
7. Tan EM, Cohen AS, Fries JF, Masi AT, McShane DJ, Rothfield NF, et al. The 1982 revised criteria for the classification of systemic lupus erythematosus. Arthritis and rheumatism. 1982;25(11):1271-7.
8. Visseren FLJ, Mach F, Smulders YM, Carballo D, Koskinas KC, Bäck M, et al. 2021 ESC Guidelines on cardiovascular disease prevention in clinical practice: Developed by the Task Force for cardiovascular disease prevention in clinical practice with representatives of the European Society of Cardiology and 12 medical societies With the special contribution of the European Association of Preventive Cardiology (EAPC). European heart journal. 2021;42(34):3227-337.
9. Mosteller RD. Simplified calculation of body-surface area. The New England journal of medicine. 1987;317(17):1098.

10. Bassingthwaighte JB, Wang CY, Chan IS. Blood-tissue exchange via transport and transformation by capillary endothelial cells. Circulation research. 1989;65(4):997-1020.
11. Kellman P, Wilson JR, Xue H, Ugander M, Arai AE. Extracellular volume fraction mapping in the myocardium, part 1: evaluation of an automated method. Journal of cardiovascular magnetic resonance : official journal of the Society for Cardiovascular Magnetic Resonance. 2012;14(1):63.
